# Diosgenin-mediated NF-κB and MAPK pathway modulation for osteoarthritis treatment: molecular mechanisms, bioavailability enhancement, and therapeutic applications

**DOI:** 10.1007/s10787-025-02102-4

**Published:** 2026-01-19

**Authors:** Rudresh Adarkar, Vijishna Lekshmi Viswambharan, Akanksha Dessai, Richard Lobo, Vamshi Krishna Tippavajhala, Vasudev Pai, Chandrashekar Kodangala Subraya, H. N. Aswatha Ram

**Affiliations:** 1 Department of Pharmacognosy, Manipal College of Pharmaceutical Sciences, Manipal Academy of Higher Education, Manipal, Karnataka 576104 India; 2 Department of Pharmaceutics, Manipal College of Pharmaceutical Sciences, Manipal Academy of Higher Education, Manipal, Karnataka 576104 India

**Keywords:** Diosgenin, Osteoarthritis, NF-κB signalling, MAPK pathway, Phytoconstituents, Bioavailability enhancement

## Abstract

The review has comprehensively examined the therapeutic potential of phytoconstituents in the treatment of arthritis with specific emphasis being given to diosgenin as a promising natural molecule. Arthritis is a significant health concern in the world, with its two most common types; Osteoarthritis and rheumatoid arthritis, becoming the causes of disability, and expensive to manage in the long run. Even though disease-modifying anti-rheumatic drugs and biologics have helped to better the patient outcomes, their side effects, high treatment cost, and inaccessibility demonstrate the necessity of safer and cheaper alternatives. The pharmacological significance of the root steroidal sapogenin, diosgenin, as a derivative of the species Dioscorea, that exhibits strong anti-inflammatory effects with clearly defined, molecular-based, mechanisms, is highlighted in this manuscript. Diosgenin suppresses cartilage destruction because of matrix metalloproteinases, the NF-kB signaling pathway as well as the major pro-inflammatory cytokines, TNF-a, IL-1b, and IL-6. It also has chondroprotective action to maintain the integrity of the extracellular matrix, increase levels of type II collagen and aggrecan, and decrease the expression of MMP-3 and MMP-13 by about 65–85% in the presence of inflammation. The challenges to clinical translation associated with formulation which are discussed in the review include the very low aqueous solubility of diosgenin (0.95 ug/mL) and oral bioavailability (< 7%). It addresses the use of the advanced delivery systems, including lipid nanoparticles, vesicular carriers and amorphous solid dispersions, which have demonstrated a 2.55-fold increase in bioavailability and also have the potential to improve therapeutic efficacy. Crucially, this review uniquely integrates these bioavailability enhancement strategies with diosgenin’s underlying molecular mechanisms, illustrating how optimised delivery systems are essential to fully unlock its therapeutic potential. Despite robust preclinical evidence supporting its anti-arthritic and chondroprotective actions, clinical investigations remain scarce, with only a few small studies and none directly evaluating diosgenin for arthritis outcomes. Overall, this review emphasises the need for well-designed clinical trials to validate the therapeutic promise of diosgenin and related phytoconstituents, paving the way for safer, accessible, and multi-targeted natural interventions for arthritis management.

**Graphical abstract:**

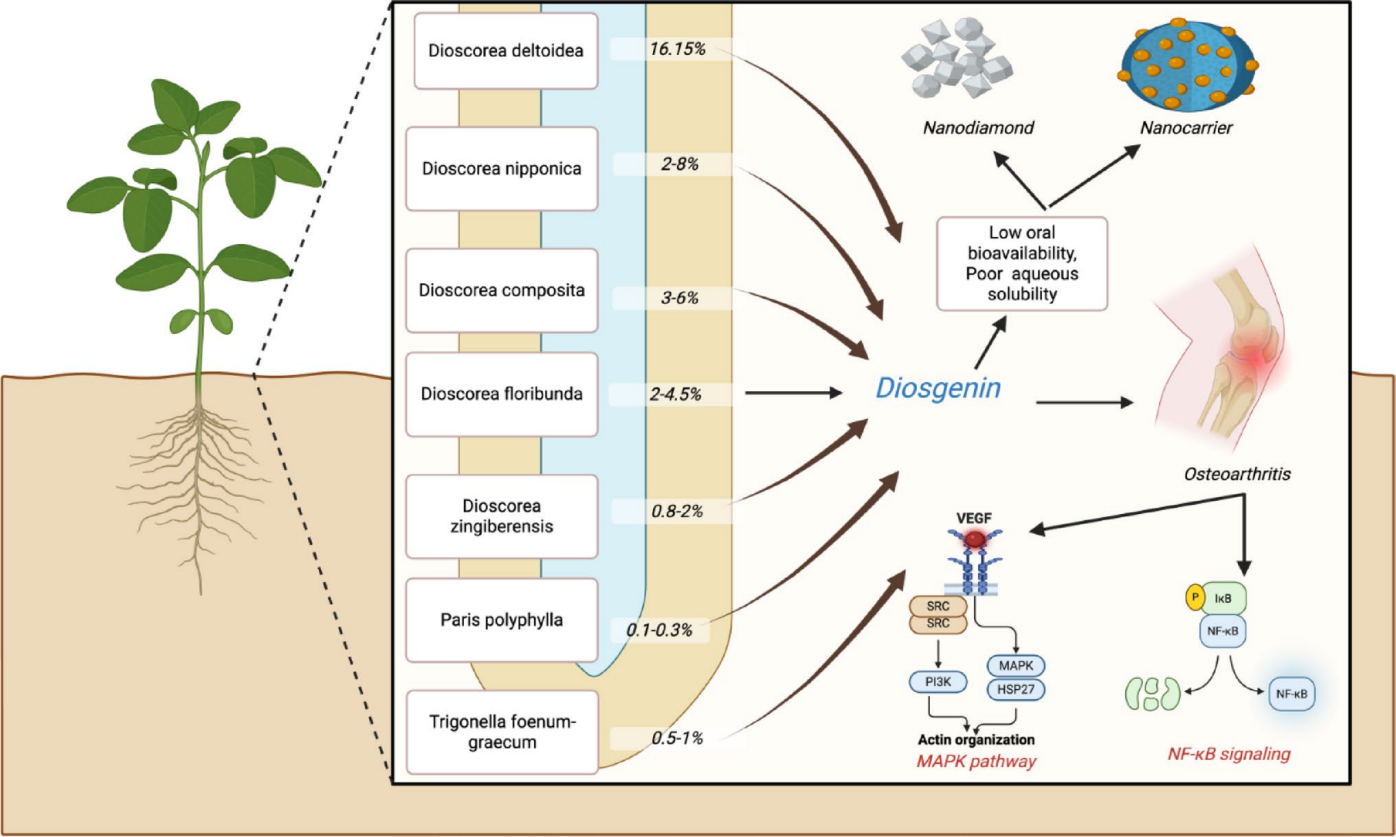

## Introduction

Arthritis is a group of more than 100 rheumatic disease conditions and continues to be one of the leading causes of global disease and disability. Among these, rheumatoid arthritis (RA) and osteoarthritis (OA) are the two most debilitating and prevalent conditions affecting the lives of millions of individuals globally. The World Health Organization (WHO) considers musculoskeletal disorders, like arthritis, as one of the leading causes of years lived with disability (YLDs), which have a significant impact on physical function, mental health, and overall quality of life (*Rheumatoid Arthritis*, n.d.). RA, an autoimmune inflammatory chronic illness, affects 0.5–1% of the world’s population, with 25 to 50 new diagnoses per 100,000 per annum(Abhishek et al. 2017; Shi et al. 2025). Untreated, RA results in irreversible joint damage, deformity, pain, and premature death. Even though improved diagnosis and treatment at disease onset have improved outcomes, many patients have co-morbidities and work disability despite treatment. Economically, RA imposes a significant burden on the healthcare system and productivity, with fees from medical care and indirect costs such as absenteeism and loss of productivity (Black et al. 2023; *Rheumatoid Arthritis: The Global Clinical Trials Landscape in 2024*, n.d.).

OA, on the other hand, is predominantly described as a degenerative disorder that most commonly occurs in older age groups. This is due to the increasing prevalence of the significant burden of obesity and inactivity taking hold. Globally, OA is a major generator of chronic pain and mobility limitation, leading to loss of autonomy and increasing dependence on long-term care (Chen et al. 2024; Steinmetz et al. 2023; Woolf 2015).

Arthritic pain results from a multifactorial interaction between inflammatory, mechanical, and neuropathic pathways, requiring multimodal treatment. Today’s approach of treatment includes NSAIDs, opioids, DMARDs, and biologics in combination with non-pharmacologic interventions such as physiotherapy and psychological support(England et al. 2023; Radu and Bungau 2021). Despite these advances, however, management of pain continues to be difficult because of the heterogeneity of pain mechanisms and responses among individual patients. Neuropathic pain elements are usually underestimated which necessitates the specific treatments and a more personalized treatment strategy (Ahmed et al. 2024). In OA, the shortcomings of present therapeutic strategies mainly emphasize symptomatic treatment without changing the disease course. NSAIDs and corticosteroids relieve pain only temporarily but don’t stop structural joint damage. The review calls for a paradigm shift towards disease-modifying osteoarthritis drugs (DMOADs) directed towards specific molecular mechanisms of cartilage breakdown, inflammation, and bone remodeling. Mobasheri et al. highlights the potential of targeted pharmacological treatment and biomarker-based treatment schedules that may result in more effective and personalized treatment of OA (Mobasheri et al. 2023).

Phytoconstituents, which are naturally occurring chemicals extracted from plants, have shown positive effects by exhibiting anti-inflammatory properties. Specifically, diosgenin has been identified as an alternative or adjunct therapeutic agent. Isolated predominantly from Dioscorea species, diosgenin is a steroidal sapogenin known to display anti-inflammatory, immunomodulatory, and antioxidant effects. Its ability to regulate the principal molecular mechanisms underlying arthritis and its favourable safety profile make it a drug candidate for the integrative treatment of arthritis (Javed et al. 2023) which will be discussed further in this review.

The pathophysiology of OA is quite interesting when we consider how the disease progresses from a small mechanical trigger to the degeneration of bone. To start with, the disease begins with as simple a mechanical trigger as stress (Jiang et al. 2023), abnormal bones such as knock knees or bow knees, ageing, and obesity (Henriques et al. 2024) joint injury, genetics Or even gender, in recent studies, it is seen that women are at a higher risk than men. These factors, with many others, act as triggering factors that trigger the first step towards OA (Segal et al. 2024).

These early recurring damages induce cellular stress and microtrauma to articular cartilage and adjacent joint tissues. Consequently, Damage-Associated Molecular Patterns (DAMPs) such as HMGB1, S100 proteins, and matrix degradation products are released from stressed or injured cells. These DAMPs will further activate pattern recognition receptors (PRRs) such as Toll-like receptors (TLRs) and also the receptor for advanced glycation end-products (RAGE) on chondrocytes and synoviocytes, triggering a sterile inflammatory response within the joint milieu (Bertheloot and Latz 2016; Palumbo et al. 2023). Furthermore, activated chondrocytes and synoviocytes induce the production of pro-inflammatory cytokines, most notably interleukin-1β (IL-1β) and tumour necrosis factor-alpha (TNF-α). These cytokines promote a change in the cartilage matrix environment towards a catabolic environment, inhibiting the production of key structural proteins such as collagen type II and aggrecan, while at the same time enhancing the production of matrix metalloproteinases (MMPs) and a disintegrin and metalloproteinase with thrombospondin motifs (ADAMTS), specifically ADAMTS-4 and ADAMTS-5. These enzymes directly catalyze the degradation of the extracellular matrix (ECM), leading to a progressive deterioration of cartilage integrity (Chen et al. 2014a, b; Wojdasiewicz et al. 2014).

With loss of the articular cartilage, the underlying subchondral bone is increasingly exposed and responds with abnormal remodeling, which is manifested as bone sclerosis, cysts, and the formation of osteophytes, osseous growths directed towards stabilizing the joint but eventually resulting in mechanical hindrance and pain. Meanwhile, bone marrow lesions may develop, adding to the nociceptive burden (Dudaric et al. 2023; Zhu et al. 2021).

Simultaneously, the inflamed synovium, perpetuated by DAMPs and cytokines, contributes to joint destruction by secreting additional pro-inflammatory mediators and proteolytic enzymes. Though the response is milder than autoimmune disorders like RA, this low-grade synovitis is sufficient to maintain chronic joint inflammation and augment tissue damage. OA pain, a hallmark symptom, results from several overlapping mechanisms: direct mechanical loading of bone and soft tissue, inflammatory mediator sensitisation of nociceptors, increased intraosseous pressure, and distension of the joint capsule. Of special interest is that pain originates from structures such as the synovium, subchondral bone, ligaments, and periarticular muscles because of the aneural nature of articular cartilage (De Roover et al. 2023; Sanchez-Lopez et al. 2022). Further, we will try to understand the underlying mechanisms of OA pathogenesis.

This article reviews studies that focus on the therapeutic potential of phytoconstituents in the treatment of arthritis, with a specific emphasis on diosgenin as a promising natural molecule. Consequently, this review critically examines diosgenin’s molecular modulation of inflammatory pathways (such as NF-kB and MMP suppression), evaluates the role of advanced delivery systems in overcoming its bioavailability challenges, and highlights the urgent need for clinical trials to bridge the gap between preclinical success and patient application.

## Methodology

A comprehensive literature search was conducted using electronic databases, including Google Scholar, PubMed, and Scopus. The search spanned publications from January 2014 to December 2025, with particular emphasis on studies retrieved from Scopus. The search terms included combinations of keywords such as “Diosgenin,” “osteoarthritis treatment,” “NF-κB signalling,” “MAPK pathway,” “anti-inflammatory mechanisms,” “bioavailability enhancement,” and “chondroprotection.” Studies were selected based on their relevance to the molecular mechanisms, bioavailability enhancement strategies, preclinical and clinical evaluation, and pharmacological modulation of NF-κB and MAPK pathways by Diosgenin for the treatment of osteoarthritis. We included peer-reviewed research articles, including original studies, reviews, and clinical trial reports, that focused on the use of diosgenin-mediated pain modulation for OA therapies. We included peer-reviewed research articles, including original studies, reviews, and clinical trial reports. Exclusion criteria included non-English publications, conference abstracts without full text, and articles not directly related to OA and diosgenin.

## Molecular mechanisms of osteoarthritis pathogenesis

### Inflammatory cascade initiation

The primary inducers of OA are mechanical stress, metabolic derangement, and age-related cellular alterations that cause microtrauma to articular cartilage. These stresses result in the release of DAMPs from injured or stressed cells, such as high-mobility group box 1 (HMGB1), S100 proteins, and matrix breakdown products. The released DAMPs engage PRRs like TLR-2, TLR-4, and attack chondrocytes and synovial cells, stimulating downstream inflammatory signaling cascades (Grässel et al. 2021; Xia et al. 2014).

### NF-κB pathway activation

The NF-κB signaling pathway is a central component of OA inflammatory pathways. In normal physiological conditions, NF-κB homodimers (commonly p50/p65) are sequestered in the cytoplasm by inhibitory proteins (IκB family). With inflammatory activation, the IκB kinase (IKK) complex phosphorylates IκBα, leading to its ubiquitination and subsequent destruction by proteasomes. This releases NF-κB from its inhibitory state, allowing it to translocate to the nucleus and induce the transcription of many pro-inflammatory genes, such as TNF-α, IL-1β, IL-6, COX-2, and inducible nitric oxide synthase (iNOS) (Choi et al. 2019).

### Matrix degradation processes

**Fig. 1:**
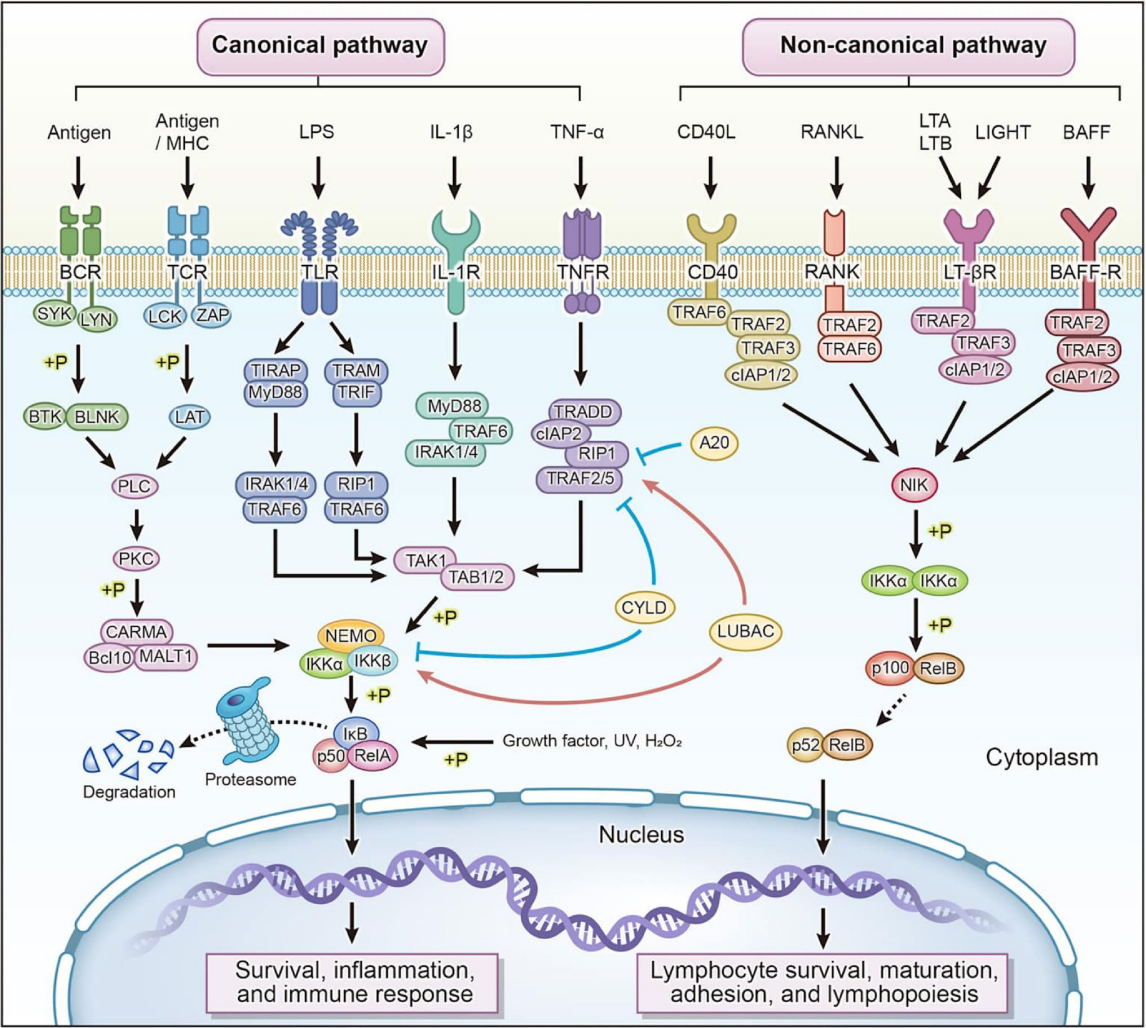
NF-κB Signaling Pathways in Inflammation

Activated chondrocytes and synovial cells release high concentrations of matrix metalloproteinases (MMPs), including MMP-1, MMP-3, and MMP-13, which sequentially cleave cartilage extracellular matrix components such as type II collagen and aggrecan. At the same time, protective matrix component synthesis is reduced, leading to a sequence of processes tending toward cartilage degradation(Mehana et al. 2019) as illustrated in Fig. 1.

The Fig. 1 illustrates the activation of NF-kB by a variety of immune receptors such as BCR, TCR, TLRs, IL-1R, TNFR, CD40, RANK, LTbR and BAFF-R activated by interdependent canonical and non-canonical pathways. Tethering of receptors results in the recruitment of adaptor proteins (MyD88, TRIF, TRAFs, RIP1/2) and upstream kinase interactions (IRAKs, TAK1, NIK), which cause activation of IKK complexes. The canonical pathway has IKK-mediated degradation of IkB that allows nuclear translocation of p50/RelA to promote the expression of inflammatory and survival genes. Non-canonical activation of IKKa by NIK leads to p100 processing to p52 that facilitates transcription by p52/RelB to mediate lymphocyte maturation and homeostasis. Pathway activity is regulated by negative regulators like A20, CYLD and LUBAC. Reproduced with permission from(Guo et al. 2024), copyright: © 2024 Nature Publications, all rights reserved.

OA, therefore, is much more than cartilage loss; it is a self-perpetuating disease process involving the whole body. It is a pathological intersection of mechanical overload, inflammatory activation through DAMPs, enzymatic tissue degradation, ineffective repair processes, and disrupted joint biomechanics. This self-perpetuating process produces chronic pain, joint stiffness, functional impairment, and ultimately disability. Treatment of OA, therefore, needs an in-depth understanding of this complex pathophysiology to direct both present management and future therapeutic interventions (Man and Mologhianu 2014; Peng et al. 2025; Yunus et al. 2020).

## The current need for phytopharmaceutical

The global OA therapeutics market is experiencing high rate growth, with projections that are indicating an increase from USD 9.91 billion in 2024 to approximately USD 24.34 billion by 2034. This expansion shows a rising demand for effective and safer OA treatments. Consumer surveys and market analyses reveal a growing inclination towards natural and plant-based therapies, driven by concerns over the side effects associated with conventional medications. This shift influences product development and clinical research in the OA treatment landscape.​ Why is there a drastic shift towards the herbal market rather than current treatment plans? The answer is safe, effective, and non-invasive medication that won’t harm the near future, keeping the disease under control.(*Osteoarthritis Therapeutics Market Size*,* Report By 2034*, n.d.)

Various plants have shown effects against OA pain and disease management, and even clinical studies are underway to discover their potential, for example, Curcumin. A meta-analysis confirmed that oral administration of curcuma longa extract significantly reduced knee pain and improved joint function in OA patients. SKCPT This herbal formulation, comprising extracts from Clematis mandshurica, Trichosanthes kirilowii, and Prunella vulgaris, has shown potential in replacing NSAIDs for knee OA treatment, with fewer long-term side effects (Hsiao et al. 2021; *SKCPT - Drug Targets*,* Indications*,* Patents - Synapse*, n.d.).

However, plants are a mix of phytoconstituents, so they need to find potent phytoconstituents that will show their effects in low concentrations. A few such phytoconstituents include Steroidal saponins (Diosgenin, Hecogenin, Sarsasapogenin), Flavonoids (Quercetin, Kaempferol), Alkaloids (Berberine, Colchicine), Terpenoids (Boswellic acid, Curcumin). The mechanism of action and targeted pathway have been summarised in Table 1. By discussing these phytoconstituents individually, we can assess the mechanism of action and the specific actions that can suppress or mitigate osteoarthritis, thereby influencing the herbal drug industry.

**Table 1:** Various phytoconstituents targeting inflammatory pathways

Class	Major source	Inflammatory pathway targeted	Mechanism of action	Study conducted	Reference
Steroidal Saponins	Dioscorea species	NF-κB, TLR4	Inhibit NF-κB activation; suppress TLR4-mediated signaling, reducing pro-inflammatory cytokines (TNF-α, IL-6, IL-1β) production	In vitro: Diosgenin (active saponin) inhibited IL-1β-induced expression of inflammatory mediators (NO, COX-2) and matrix-degrading enzymes (MMP-3, MMP-13) in human OA chondrocytes. In vivo: Oral administration of Diosgenin significantly attenuated cartilage deterioration and subchondral bone changes in rat OA models by suppressing the NF-κB signaling pathway.	Guoying et al. (2021)
Flavonoids	Citrus fruits, tea, berries	NF-κB, MAPK	Inhibit phosphorylation of IκB, downregulate NF-κB; suppress MAPK pathways, reducing COX-2, iNOS, cytokine production	**Clinical**: Green tea polyphenols (rich in EGCG) have been shown to reduce pain and improve WOMAC scores in knee OA patients. **In vivo & In vitro**: Flavonoids like Naringin (Citrus) and EGCG (Tea) protected against cartilage loss in murine OA models and suppressed MAPK/NF-κB phosphorylation in IL-1β-stimulated chondrocytes.	Panche et al. (2016)
Alkaloids	Berberis species, Piper species	NF-κB, JAK/STAT	Block NF-κB DNA binding; inhibit JAK/STAT phosphorylation; reduce inflammatory mediators and cell adhesion molecules	In vivo: Berberine (*Berberis*) and Piperine (*Piper*) prevented cartilage degradation and reduced pain behavior in MIA-induced and surgical OA rat/mouse models. In vitro: These alkaloids inhibited the nuclear translocation of NF-κB and suppressed STAT3 phosphorylation in human chondrocytes, reducing the release of IL-6 and TNF-α.	Haftcheshmeh et al. (2022)
Terpenes	Essential oils (e.g., limonene, pinene)	NF-κB, COX-2, LOX	Inhibit NF-κB nuclear translocation; reduce COX-2, LOX expression; decrease NO and prostaglandin synthesis	In vitro: Pinene (a major terpene) significantly inhibited IL-1B induced NF-κB activation and reduced the production of catabolic enzymes (MMP-1, MMP-13) and NO in human OA chondrocytes. In vivo: Oral administration of terpenes (e.g., Limonene, Myrcene) in arthritis animal models (CFA or collagen-induced) reduced joint edema, mechanical hypersensitivity, and cytokine levels (TNF-α, IL-6). **Clinical**: Randomized controlled trials have shown that topical application of terpene-rich essential oils (e.g., Hemp seed oil containing terpenes, or Lavender/Rosemary blends) significantly reduced knee pain (WOMAC scores) and stiffness in OA patients compared to placebo.	de Lavor et al. (2018), Hashemzadeh et al. (2020), Rufino et al. (2014)

## Steroidal saponins and their role in inflammation

**Fig. 2:**
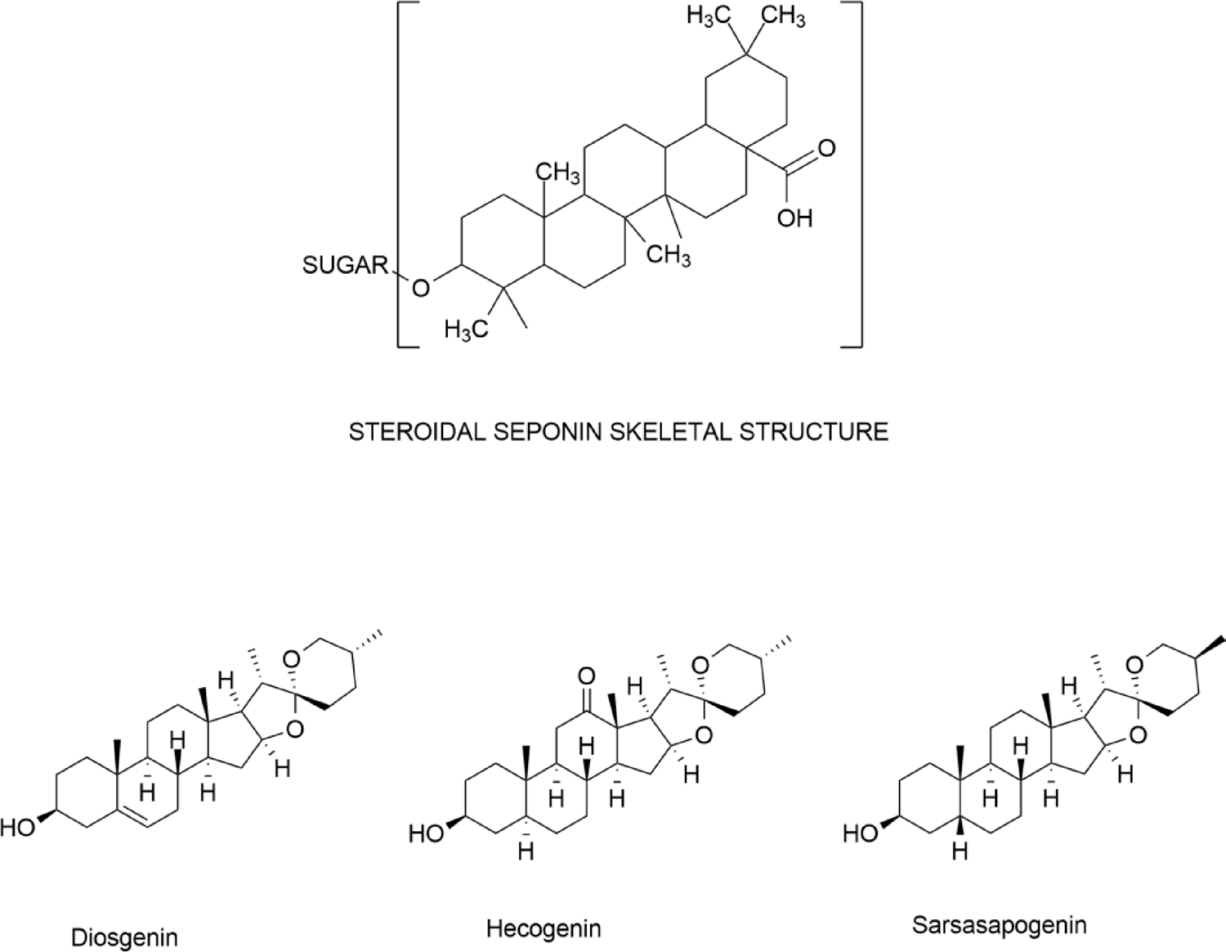
Skeletal structure of steroidal saponin along with key steroidal saponins like Hecogenin, Sarasa sapogenin, and Diosgenin

Steroidal saponins constitute a unique class of naturally occurring glycosides with a hydrophobic steroidal aglycone (sapogenin) attached to hydrophilic sugar chains as shown in the Fig. 2, which contributes to their amphiphilic nature that endows them with surfactant as well as biological membrane-interacting activity (R. Chen et al. 2023a, b). Structurally, the aglycone core is a cyclopentanoperhydrophenanthrene ring system, as in steroids, and can also include additional ring systems like spiroketals in spirostanol saponins or furan rings in furostanol saponins. Glycosidic linkage at the C-3 hydroxyl position is typical, and glucose, rhamnose, galactose, or xylose determines their solubility and pharmacokinetics (Guo et al. 2020). The compounds are found in monocot plant families like Liliaceae, Dioscoreaceae, and Asparagaceae, and occur in a variety of plant parts like roots, rhizomes, and leaves, where they serve vital defense roles against herbivores and microbial pathogens in virtue of bitterness as well as cell-membrane-disrupting activity (Li et al. 2023). Steroidal saponins are biosynthesized from the mevalonate pathway to synthesize isopentenyl diphosphate (IPP), which dimerizes to give squalene. The latter is epoxidized and cyclized to give cycloartenol, the plant sterol precursor, which is enzymatically converted to cholesterol. Cholesterol is subjected to characteristic oxidations, typically involving cytochrome P450s, to give steroidal sapogenins like diosgenin, which are glycosylated by glycosyltransferases to give the intact saponin molecule (Chen et al. 2023a, b). Their amphiphilic nature allows them to form stable foams, solubilize cholesterol, and permeabilize biological membranes, properties utilized in pharmaceutical formulations as natural emulsifiers or adjuvants (Timilsena et al. 2023). Besides these physicochemical properties, steroidal saponins also have a biological role in plant signaling and environmental stress responses. Their biochemical sophistication, biosynthetic specificity, and wide-spectrum bioactivity render them an interesting area of investigation in natural product chemistry and pharmacognosy, especially for their possible functions in immunomodulation, anti-inflammatory activity, and as natural therapeutic agents in chronic diseases like OA (Cui et al. 2024).

**Fig. 3:**
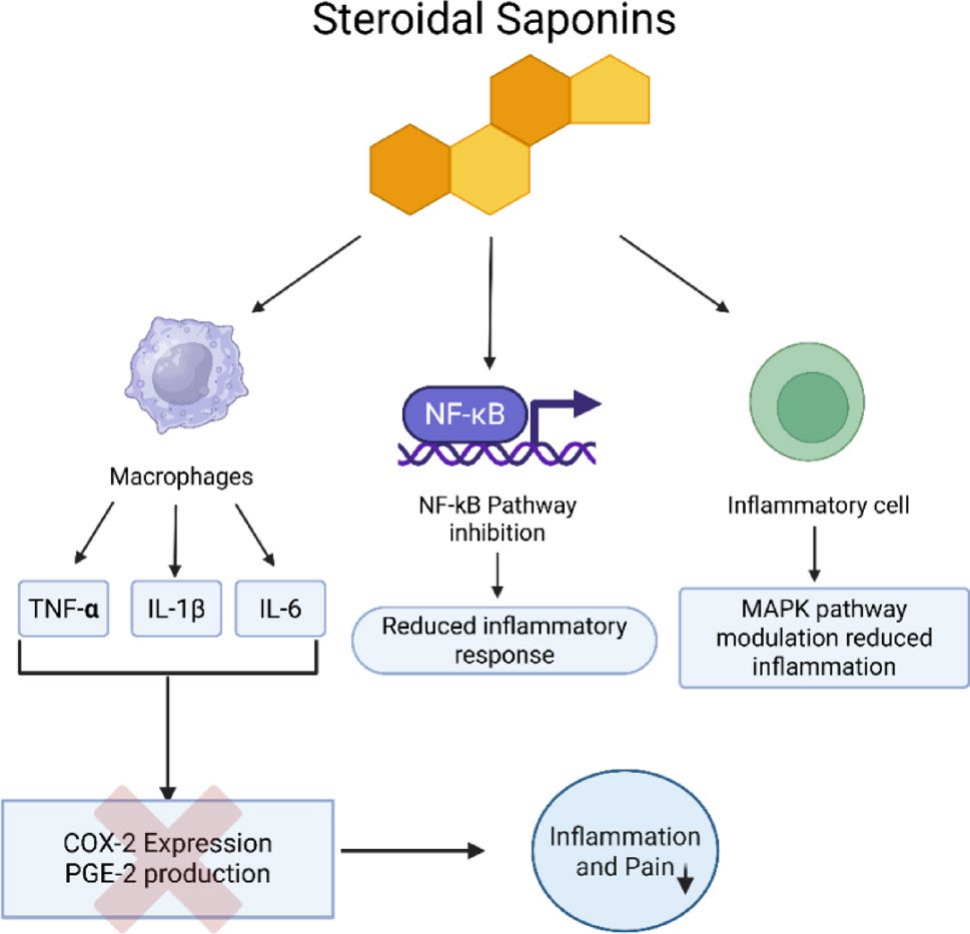
Mechanism of action of steroids with different pathways suppressing the pain

Steroidal saponins are a structurally heterogeneous group of secondary metabolites distributed widely among marine organisms like starfish and sea cucumbers, and widely distributed in the plant kingdom, especially in such families as Alliaceae, Asparagaceae, Dioscoreaceae, Scrophulariaceae, Bromeliaceae, Amaryllidaceae, Palmaceae, and Liliaceae. They are also present in food crops such as Dioscorea japonica (yam), Allium species (garlic and onion), Trigonella foenum-graecum (fenugreek), Yucca schidigera (Mojave yucca), and Panax species (ginseng) (Passos et al. 2022). Steroidal saponins gained pharmaceutical interest since the 1950 s, initially as steroidal hormone synthesis starting materials, as attested by progesterone synthesis from diosgenin, significantly improving oral contraceptives (Bouabdallah et al. 2023). Recently, their therapeutic potential against inflammatory diseases has gained considerable attention. Steroidal saponins have multiple mechanisms of action against inflammation and inhibit major pro-inflammatory cytokines, including tumor necrosis factor-alpha (TNF-α), interleukin-1 beta (IL-1β), and interleukin-6 (IL-6) (Passos et al. 2022). These cytokines are central in neuroendocrine disturbances and symptoms of various inflammatory and autoimmune diseases, generally called “sickness behaviors.” Steroidal saponins inhibit cytokine production, modulate macrophage activity, and disrupt central inflammatory signalling cascades, notably the arachidonic acid pathway, leading to inhibited cyclooxygenase-2 (COX-2) and prostaglandin E2 (PGE2) production. Furthermore, these compounds effectively modulate the NF-κB pathway, a master regulator of inflammation implicated in chronic diseases. By inhibiting NF-κB activation, steroidal saponins block the transcription of numerous pro-inflammatory genes, offering a promising avenue for controlling inflammation. Finally, modulation of the MAPK (mitogen-activated protein kinase) pathway by steroidal saponins is another central anti-inflammatory mechanism. The MAPK signalling pathway is a key mediator between extrinsic signals and cellular response, which has been illustrated in Fig. 3. Thus, blockading the MAPK signaling pathway can inhibit excessive inflammatory reactions, making saponins promising lead compounds in the treatment of autoimmune and chronic inflammatory disorders (Wijesekara et al. 2024). Corticosteroids are widely used to suppress inflammation but have severe adverse effects upon long-term administration, such as immunosuppression. Steroidal saponins, which have plant origins and are easily available, could present a safer alternative, with fewer adverse effects. Besides their anti-inflammatory activity, steroidal saponins also possess antioxidant activity, which further adds to their therapeutic potential by mitigating oxidative stress-a predominant aspect of chronic inflammation. Though promising preclinical findings that indicate their capacity to modulate inflammatory mediators and signaling pathways provide encouraging evidence, challenges remain, particularly the pharmacokinetics, bioavailability, and specific molecular mechanisms associated with individual saponins. More clinical trials must prove their efficacy and safety in humans. However, the evidence puts steroidal saponins in place as potentially valuable bioactive molecules capable of modulating inflammation via multiple molecular targets, particularly through cytokine modulation and NF-κB, TLR4, and MAPK pathways inhibition which is shown in Fig. 3. The results provide evidence for the significance of the traditional medicinal properties of saponin rich plants and an appropriate platform for future drug discovery initiatives aimed at treating inflammation and pain via plant-derived therapeutic agents.(Passos et al. 2022).

### Role of diosgenin in inflammation

**Fig. 4:**
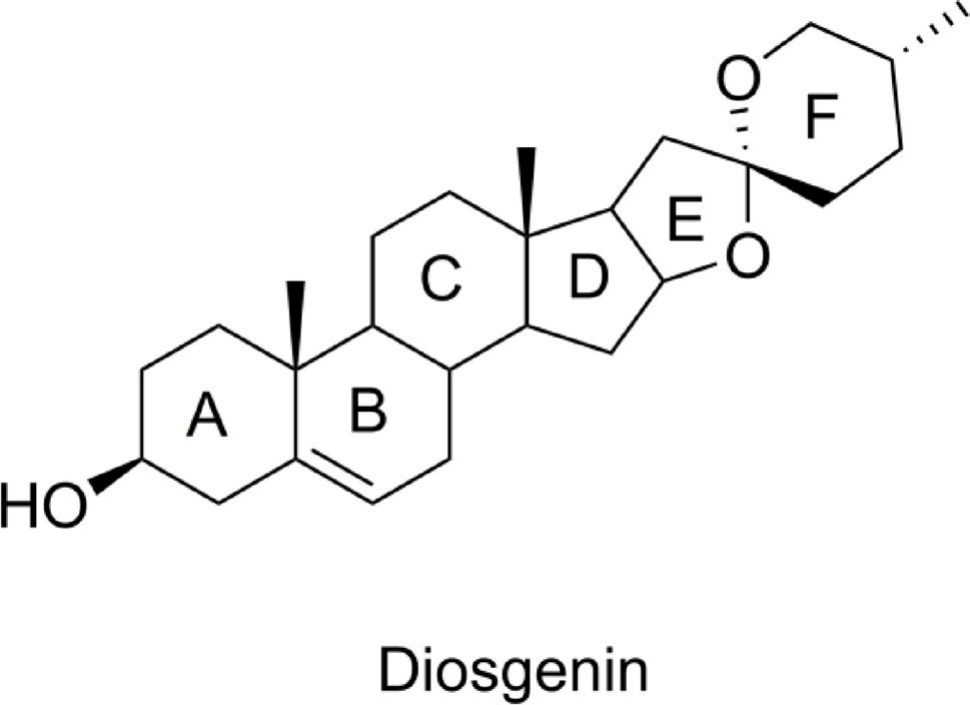
Skeletal structure of diosgenin for analysing its structure activity relationship

Diosgenin, a steroidal sapogenin primarily sourced from *Dioscorea* species, has garnered significant interest due to its potent anti-inflammatory effects across various medical conditions, including Alzheimer’s disease, hyperlipidemia, type 2 diabetes, and atherosclerosis(ur-Rehman et al. 2024) (Adarkar et al. 2025). Skeletal structure of Diosgenin is as shown in the Fig. 4. Notably, studies focusing on human OA chondrocytes have demonstrated that diosgenin effectively reduces the synthesis of interleukin-1 (IL-1)-induced pro-inflammatory mediators such as prostaglandin E2 (PGE2) and nitric oxide (NO) (Wang et al. 2015a). Furthermore, diosgenin inhibits the degradation of inhibitor kappa B-alpha (IκB-α) and the expression of matrix metalloproteinases (MMP-3 and MMP-13), as well as other IL-1-modulated proteins, including cyclooxygenase-2 (COX-2) and inducible nitric oxide synthase (iNOS). Beyond its effect on joint health, diosgenin plays a role in bone homeostasis by modulating long non-coding RNAs (lncRNAs), reducing the receptor activator of nuclear factor-kappa B ligand (RANKL) to osteoprotegerin (OPG) ratio, which is crucial for preventing osteoclast-mediated bone resorption (Wang et al. 2015a; Zhang et al. 2018). The anti-inflammatory mechanism of diosgenin is primarily mediated through the nuclear factor kappa-light-chain-enhancer of activated B cells (NF-κB) signaling pathway and glucocorticoid receptor (GR) activation. This was validated in an ovalbumen (OVA)-induced asthmatic mouse model, where diosgenin not only reduced NF-κB activation but also increased the expression of glucocorticoid-induced leucine zipper (GILZ), secretory leukocyte protease inhibitor (SLPI), and mitogen-activated protein kinase phosphatase-1 (MKP-1). As a result, these upregulated proteins reduced inflammation in the lower respiratory tract by suppressing the expression of heat shock protein 70 (HSP70). Additionally, mechanistic insights have shown that activated glucocorticoid receptors bind with CREB-binding protein (CBP) and cAMP response element-binding protein (CREB), which results in a downregulation of NF-κB expression and the subsequent suppression of inflammatory cytokines like interleukin-6 (IL-6), tumor necrosis factor-alpha (TNF-α), and interleukin-1 beta (IL-1β). Chronic inflammation is effectively reduced by this cascade (Junchao et al. 2016).

Diosgenin also has anti-fibrotic properties. It is suggested that diosgenin’s anti-fibrotic effect is mediated through the TGF-β1/Smad signaling pathway because it inhibits cell proliferation in hepatic stellate cells (HSCs) and decreases the secretion of transforming growth factor-beta receptors (TGF-βRI and RII), alpha-smooth muscle actin (α-SMA), collagen I, and phosphorylated Smad3 (p-Smad3). These various bioactivities highlight diosgenin’s therapeutic potential in the treatment of conditions linked to inflammation (Xie et al. 2015).

Studies of the structure-activity relationship (SAR) have shed important light on the functional groups necessary for the bioactivity of diosgenin. The role of the 5,6-double bond and the presence of a heterosugar moiety in mediating apoptosis and anticancer effects has been emphasized by molecular modeling research. Furthermore, the pharmacological characteristics of diosgenin are strongly influenced by structural alterations at C-5 and C-25 (Mondal 2023). It’s interesting to note that, in contrast to the native hydroxyl group, acetylation at the A-ring (more especially, at the C-3 position) may strengthen its anti-inflammatory properties(Zhang et al. 2022) Diosgenin’s permeability, solubility, neuroprotective activity, and toxicity have all improved as a result of chemical changes like the addition of amide, ester, amine, and amino acid moieties at C-3 (Ren et al. 2023). Strong neuroprotective agents have been produced by derivatives created by saturating the C-5/C-6 double bond and converting the C-3 hydroxy into a phenolic hydroxyl group. Moreover, there has been substantial anti-tumor activity shown when triazole moieties such as 4-fluorophenyl, phenyl, 4-methoxyphenyl, 3,4,5-trimethoxyphenyl, or 2-pyridinyl are added at C-6 (Huang et al. 2025). Other novel modifications have demonstrated cytotoxic effects against cancer cell lines like K562 and A549, including amino acid esters and 1,3,4-oxadiazole derivatives at C-26 (El-Masry et al. 2022). Significant antiproliferative, apoptotic, and anti-inflammatory properties have been demonstrated by diosgenin spirostane oximes with hydroxyamino groups at either the F-ring or the B-ring. The C-3 position of the A-ring is still the most frequently modified site; esterification and the addition of amino acids have produced derivatives with a variety of bioactivities (Sánchez-Sánchez et al. 2016). Notably, Yang et al.‘s carbamate derivatives have demonstrated dual anti-inflammatory and anti-Alzheimer’s properties, thereby broadening the therapeutic potential of diosgenin. Huang et al. (2022) summary of key derivatives and their bioactives are being demonstracted in Table 2.

**Table 2:** Structure activity relationship of Diosgenin and its impact on bioactivity

Structural site/ring	Chemical modification/group	Impact on bioactivity	Reference
General Structure	5,6-Double Bond & Heterosugar Moiety	Critical for mediating apoptosis and anticancer effects.	Molecular modeling research
C-5 & C-25	Structural alterations	Strongly influences general pharmacological characteristics.	Mondal (2023)
A-Ring (C-3 Position)	Acetylation	May strengthen anti-inflammatory properties compared to the native hydroxyl group.	Zhang et al. (2022)
C-3 Position	Addition of Amide, Ester, Amine, or Amino Acid moieties	Improves permeability, solubility, and neuroprotective activity while reducing toxicity.	Ren et al. (2023)
C-3 & C-5/C-6	Saturation of 5,6-double bond + conversion of C-3 hydroxy to Phenolic Hydroxyl	Creates derivatives that act as strong neuroprotective agents.	Ren et al. (2023)
C-6 Position	Addition of Triazole moieties (e.g., 4-fluorophenyl, 2-pyridinyl)	Demonstrates substantial anti-tumor activity.	Huang et al. (2025)
C-26 Position	Amino Acid Esters & 1,3,4-Oxadiazole derivatives	Shows cytotoxic effects against specific cancer cell lines (e.g., K562, A549).	El-Masry et al. (2022)
F-Ring or B-Ring	Spirostane Oximes with Hydroxyamino groups	Demonstrates significant antiproliferative, apoptotic, and anti-inflammatory properties.	Sánchez-Sánchez et al. (2016)
General/C-3 (Implied)	Carbamate derivatives	Exhibits dual anti-inflammatory and anti-Alzheimer’s properties.	Huang et al. (2022)

Despite diosgenin’s great therapeutic potential, its low bioavailability makes it difficult to use in clinical settings. Rats’ oral bioavailability of diosgenin has been reported to be less than 7%, indicating low intestinal absorption (ur-Rehman et al. 2024). Its low aqueous solubility (about 0.95 µg/mL) and strong hydrophobicity are the main causes of its reduced bioavailability. To increase its absorption, a number of methods have been investigated, including forming diosgenin into microemulsions or adding cyclodextrins, polyethylene glycol, and chitosans. According to pharmacokinetic studies, diosgenin reaches a serum concentration of roughly one µg/mL in humans following oral administration of 3 g/day for four weeks. Radiolabeled diosgenin showed quick excretion in animal models, with 99% of radioactivity removed through feces and 1% through urine, mostly in less than a day (Semwal et al. 2022). Metabolites such as monohydroxylated derivatives (e.g., smilagenin and 7-hydroxydiosgenin isomer) have been identified in the bile of dogs and caecal contents of rats, indicating significant biotransformation by intestinal microflora (Salunkhe et al. 2021).

Regarding clinical relevance, four notable clinical trials have investigated diosgenin-containing extracts. These include a study in Japan where diosgenin-rich yam extract significantly improved cognitive function over 12 weeks, and a crossover trial in healthy menopausal women where wild yam cream showed no serious side effects. Additionally, diosgenin combined with nuciferine was evaluated for improving ejaculation control in men with premature ejaculation, and a multi-nutrient supplement containing diosgenin demonstrated therapeutic potential in other conditions (Tohda et al. 2017).

The compelling preclinical and clinical data highlight diosgenin’s multifaceted anti-inflammatory mechanisms, its SAR-based optimization for enhanced bioactivity, and the need for improved formulations to overcome bioavailability barriers. These attributes position diosgenin as a promising candidate for future development in managing chronic inflammatory diseases. Diosgenin has also been seen to modulate the pain receptors, but the major drawback of using it topically for skin delivery has always been its irritability. These can be overcome by using novel drug delivery targets to enhance the skin permeation and penetration (*Diosgenin | C27H42O3 | CID 99474 - PubChem*, n.d.-a).

### Biosynthetic pathway and plant sources

**Fig. 5:**
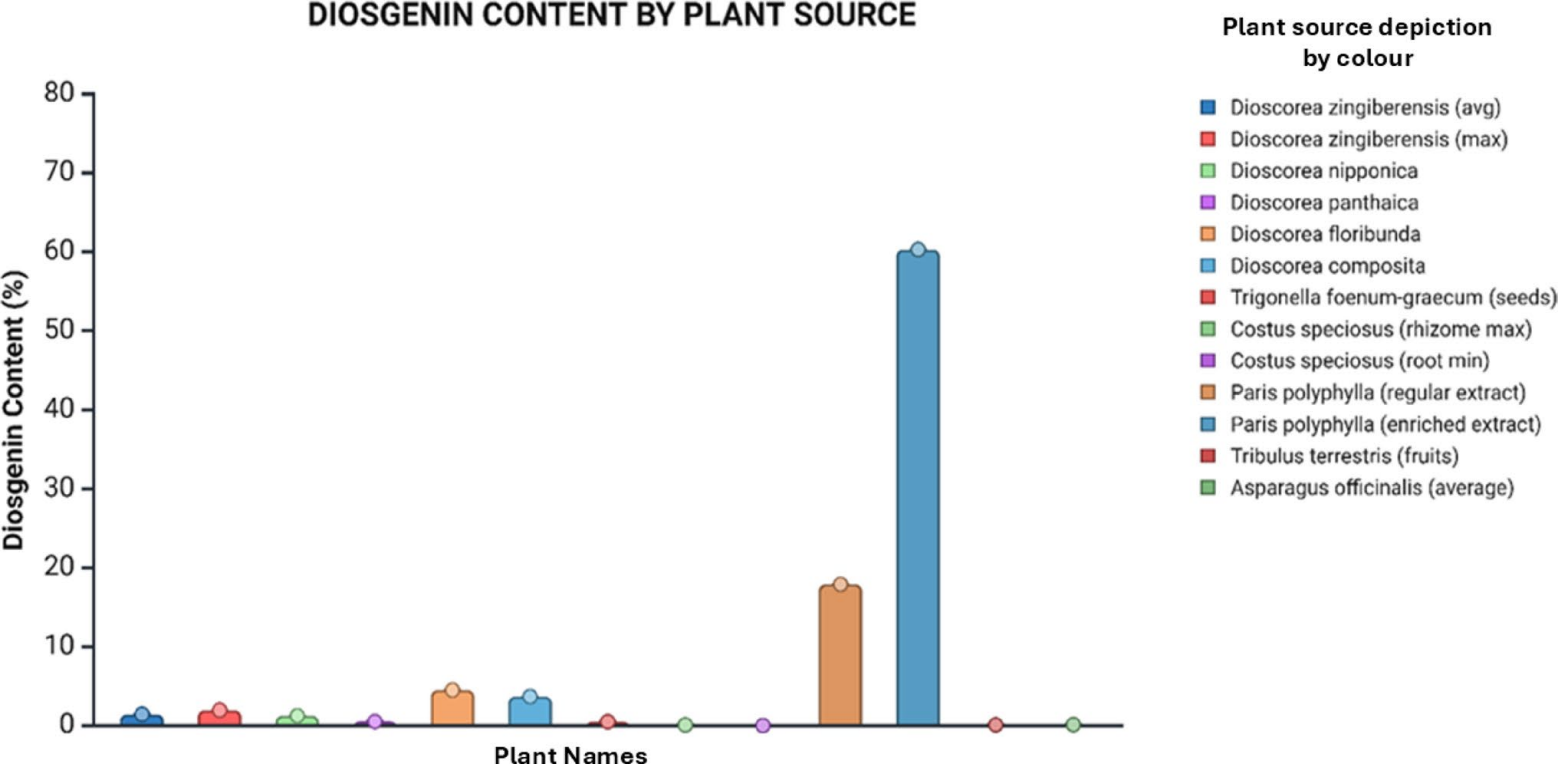
Comparative analysis of diosgenin content (%) across different plant sources, highlighting species-specific variations, with the highest yield observed in Paris polyphylla (enriched extract), followed by Paris polyphylla (regular extract) and Dioscorea floribunda. Figure illustrated using Biorender.com

Diosgenin biosynthesis follows the mevalonate (MVA) pathway, beginning with acetyl-CoA and progressing through key intermediates including mevalonate, isopentenyl pyrophosphate (IPP), farnesyl pyrophosphate (FPP), and squalene. The path culminates in cholesterol formation, which undergoes specific hydroxylations at C-22, C-16, and C-26 positions by cytochrome P450 enzymes (particularly CYP90B71, CYP90G6, and CYP94D144) to produce diosgenin.(Guerra et al. 2021; Zhou et al. 2022). Dioscorea species represent the richest natural sources of diosgenin, with *D. zingiberensis* containing up to 16.15% diosgenin by dry weight (Yang et al. 2025), Other plant sources of diosgenin have been summarized in Fig. 5. Other significant sources include *D. nipponica* (2–8%) (Ou-Yang et al. 2018), D. *composita* (3–6%), and various other botanical families including Fabaceae (*Trigonella foenum-graecum*), Melanthiaceae (*Paris polyphylla*), and Smilacaceae (*Smilax china*) (Li et al. 2022; Nazir et al. 2021).

### Extraction and analysis methods

An important consideration for research applications and possible therapeutic development is the optimization of diosgenin extraction. Traditional extraction techniques have greatly changed from modern approaches:

### Advanced extraction technologies

These advantages have made microwave-assisted extraction (MAE) the method of choice for obtaining higher yields in the range of 1.2–1.8% w/w, with shorter extraction times (0.5–2 h) and purer extracts (90–96%). Targeted microwave energy application improves mass-transfer efficiency by cell wall disruption. (Chery et al. 2020).

Supercritical Fluid Extraction (SFE) using CO_2_ yields extracts of the highest purity (92–98%), and other organic solvents are also removed. Although the yields are average (0.6–1.0.6.0% w/w), this is a versatile method in pharmaceutical applications because of higher purity. (Arya et al. 2023; *[Research on the Application of Supercritical Fluid Extraction of Diosgenin of Smilax China] - PubMed*, n.d.).

By enzymatically breaking down plant cell walls, Enzyme-Assisted Extraction employing cellulase and pectinase exhibits the highest yields (1.5–2.2% w/w). While maintaining high levels of purity, this green technology approach has benefits for the environment (Arya et al. 2023).

### Anti-inflammatory mechanisms of Diosgenin

#### NF-κB pathway Inhibition

Diosgenin shows a potent inhibitory effect on NF-κB signalling through multiple mechanisms. Primary mechanisms include:

1. IκBα Stabilization: Diosgenin keeps NF-κB sequestered in the cytoplasm by preventing IκBα phosphorylation and subsequent degradation (Wang et al. 2015b).
2. IKK Complex Inhibition: By directly suppressing IKK activity, the substance stops the degradation cascade from being activated upstream (Shishodia and Aggarwal 2006).
3. p65 Nuclear Translocation Blockade: Diosgenin prevents the p65 subunit from nuclear translocating, even in the presence of NF-κB liberation (Song et al. 2012).
4. DNA Binding Interference: Diosgenin may directly disrupt the interactions between NF-κB and DNA, which would lower transcriptional activity.(Shishodia and Aggarwal 2006).

#### Cytokine suppression

TNF-α Inhibition: Diosgenin predominantly decreases the transcriptional and translational levels of TNF-α production. In vitro studies demonstrate that the treatment of diosgenin at 10–50 µM decreases the level of TNF-α by 60–80%. The mechanism for such activity involves NF-κB inhibition and other post-transcriptional processes affecting mRNA stability. IL-1β Suppression: The compound, with IC₅₀ values between 15 and 35 µM, efficiently diminishes IL-1β-induced inflammatory responses in chondrocytes. Such suppression is related to decreased expression of downstream inflammatory mediators like COX-2, iNOS, and other MMPs. IL-6 Modulation: Diosgenin has an inhibitory effect on IL-6 production, adding to its anti-inflammatory action, although its research is less complete.(Cong et al. 2024).

#### COX-2 and prostanoid pathway modulation

Diosgenin is effective in suppressing the expression of COX-2 by nearly a 100% through activation of the glucocorticoid receptor (GR); inhibition of the NF-kB. It reduces production of prostaglandin E 2 (PGE 2) and associated inflammatory reactions through this twofold process. Besides, the compound inhibits microsomal prostaglandin E synthase-1 (mPGES-1)-mediated prostanoid-mediated inflammation. (Tsukayama et al. 2021).

#### Chondroprotective mechanisms

Diosgenin exhibits its potent inhibitory effects on key Matrix Metalloproteinase Inhibition involved in cartilage degradation:

1. MMP-3 (Stromelysin-1): Reduced by 65–75% in IL-1β-stimulated chondrocytes.
2. MMP-13 (Collagenase-3): Decreased by 70–85% in inflammatory conditions.
3. MMP-1 (Interstitial collagenase): Moderate inhibition (40–60% reduction).

These effects occur through direct enzymatic inhibition and reduced gene transcription via NF-κB pathway suppression (Kang et al. 2018).

#### Extracellular matrix preservation

Beyond MMP inhibition, diosgenin promotes protective matrix synthesis by enhancing the production of:

1. Type II Collagen: 40–60% increase in synthesis rates.
2. Aggrecan: Enhanced proteoglycan production.
3. Decorin and Biglycan: Improved small leucine-rich proteoglycan expression.

This anabolic activity helps maintain cartilage integrity and functional properties (Chery et al. 2020).

### Methods for Diosgenin analysis

The gold standard for diosgenin analysis is Ultra-Performance Liquid Chromatography-Mass Spectrometry (UPLC-MS/MS), which has remarkable specificity and sensitivity (LOD: 0.05 µg/mL). The technique allows diosgenin and related saponins to be quantified simultaneously in complex matrices (Liu et al. 2022).

Excellent sensitivity (LOD: 0.1 µg/mL) is provided by gas chromatography-mass spectrometry (GC–MS), which is especially useful for analyzing sapogenins after acid hydrolysis. By using distinctive fragmentation patterns, this method allows for structural confirmation(Pawar, n.d.) as shown in Table 3.

**Table 3:** Methods reported for analysis of Diosgenin

Analytical method	Detection wavelength/conditions	Mobile phase/solvent system	Retention time (min)	LOD/LOQ (µg/mL)	References
HPLC-UV	UV 203 nm	Acetonitrile: Water (90:10 v/v)	15–20	0.1/0.3	Priyadarshini et al. (2022)
HPTLC	366 nm (after derivatization)	Toluene: Ethyl acetate: Formic acid (5:4:1)	Rf = 0.69Â±0.02	0.05/0.15	Laila et al. (2014; Mendhulkar (2016)
UPLC-MS/MS	MS/MS detection	Gradient acetonitrile-water	08–12	0.01/0.03	Liu et al. (2022)
GC-MS	Electron impact ionization	Helium carrier gas	25–30	0.5/1.5	Jesus et al. (2016)
Spectrophotometry	450 nm (after anisaldehyde reagent)	Various organic solvents	N/A	1.0/3.0	Raina and Misra (2020)
ELSD Detection	Evaporative light scattering	Hexane: Ethyl acetate systems	12–18	0.2/0.6	Jesus et al. (2016)

### Challenges in Diosgenin delivery

Pronounced pharmacokinetic limitations essentially curtail diosgenin’s therapeutic potential. Diosgenin exhibits very slow dissolution in biological fluids, with an aqueous solubility of only 0.7 ng/mL. This poor dissolution is directly related to inferior bioavailability and diminished therapeutic effectiveness. Diosgenin has a half-life of 11.3 min in S9 fractions, which suggests rapid phase II metabolism. As such, this rapid clearance significantly reduces systemic exposure upon oral administration. Moreover, diosgenin exhibits poor cellular uptake and bioavailability due to its nature as a substrate for P-glycoprotein efflux transporters (Biswas et al. 2024; Chen et al. 2014a, b; Okawara et al. 2014). Due to these limitations, various novel techniques can be adopted to improve its properties.

The effective delivery of Diosgenin is significantly hindered by its physicochemical properties and safety profile during formulation. Diosgenin is a lipophilic molecule with a high molecular weight (414.62 g/mol) and a log P value of approximately 5.7, resulting in poor aqueous solubility and challenges in achieving adequate skin permeation without the use of enhancers(*Diosgenin | C27H42O3 | CID 99474 - PubChem*, n.d.-b).

A critical challenge is its classification as a skin and eye irritant (GHS Category 2), which complicates the development of high-concentration topical formulations. While Diosgenin exhibits anti-inflammatory effects therapeutically, the raw active pharmaceutical ingredient (API) can cause contact dermatitis or local irritation, necessitating careful selection of non-irritating vehicles. Furthermore, its rigid crystalline structure requires solubilizers that may exacerbate skin sensitivity, creating a delicate balance between effective permeation and cutaneous safety (*Safety Data Sheet Acc. to OSHA HCS*, n.d.; Singh Chaudhary et al. 2024).

### Innovative delivery solutions

Nano-formulation Approaches have been developed to curb diosgenin’s limitations(Dessai et al. 2024; Dhanush Gowda et al. 2025).Aslam et al. made solid lipid nanoparticles (SLN) that were sustained release and had a bioavailability of 28 h duration and was highly promising in terms of delivering diosgenin. Optimised formulations were found to have particle sizes of 170–180 nm and entrapment efficiencies of over 70%. (Aslam et al. 2024). Utilizing polymeric carriers such as Soluplus, amorphous solid dispersions achieve a remarkable five-fold increase in bioavailability. By fostering advantageous drug-polymer interactions, these carriers promote dissolution and inhibit crystallization (Liu et al. 2020).

As demonstrated by Zhe et al., Nanocrystal technology preserves drug stability while increasing bioavailability by 2.55. Absorption and dissolution rates were significantly increased by the smaller particle size of 229 nm (Zhe Liu et al. 2017).

### Transdermal and targeted delivery

**Fig. 6:**
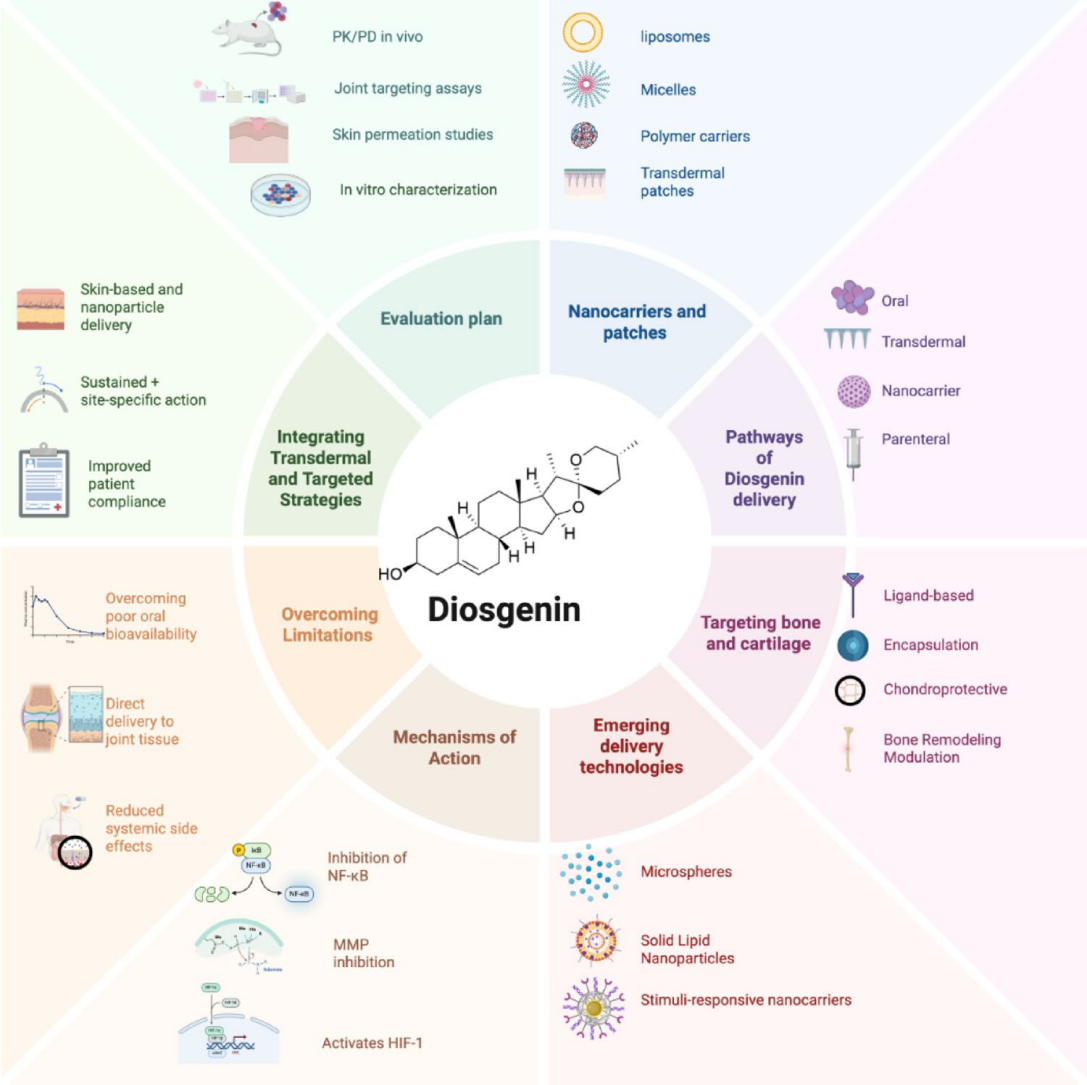
Schematic overview of innovative delivery strategies and therapeutic applications of Diosgenin. (Figure generated using BioRender.com)

With their improved skin penetration and localized delivery, transdermal systems hold special promise for the treatment of OA. High drug loading is achieved with minimal systemic exposure thanks to these ultra-deformable vesicles (Abdellatif et al. 2023; Aldawsari et al. 2024; Gharat et al. 2025). Selective delivery to diseased cartilage is made possible by targeted nanocarriers that use ligands specific to joints. Peptide-conjugated C5-24 nanoparticles show improved retention in injured joints while lowering side effects (Zhao et al. 2024). Figure 6 illustrates a comprehensive approach to enhancing Diosgenin efficacy, encompassing nanocarrier and transdermal formulations, specific administration pathways, and targeted delivery to bone and cartilage. It further details the mechanisms of action, strategies for overcoming pharmacokinetic limitations, and the evaluation plan for these novel delivery systems.

### Clinical translation

There are still few diosgenin clinical trials available, despite a wealth of preclinical data. The crucial necessity of improved delivery systems was highlighted by a noteworthy human study that administered 3 g/day orally for 4 weeks but failed to produce detectable serum levels (> 1 mg/mL) (Cai et al. 2020; ur-Rehman et al. 2024)as reported in Table 3.

As reported in Table 4, this clinical landscape highlights a significant research gap; existing human trials are not only sparse but also entirely lack investigations into osteoarthritis, further underscoring the need to bridge the divide between preclinical efficacy and clinical reality.

**Table 4:** Summary of clinical interventions and pilot studies involving Diosgenin

NCT number/registration number	Study design	Population	Intervention	Duration	Primary Endpoint	Key findings	Arthritis relevance	Refernces
UMIN000021151	Placebo-controlled, randomized, double-blind, crossover study	Healthy adults (n = unclear)	Diosgenin-rich yam extract	12 weeks	Cognitive function	Significant improvement in cognitive function	None (cognitive study)	Tohda et al. (2017)
Conducted before mandatory registration (2005)	Randomized controlled trial	Healthy menopausal women (n = unclear)	Wild yam cream (topical)	Unclear	Menopausal symptoms, lipids, sex hormones	No serious side effects	Limited (hormonal effects may influence joint health)	Komesaroff et al. (2001)
Pilot study	Pilot study	Men with premature ejaculation	Diallyl thiosulfinate + nuciferine + diosgenin	Unclear	Ejaculation control	Improved ejaculation control	None (sexual health study)	Cai et al. (2018)
Pilot study	Clinical trial (details not specified)	Unclear	Multi-nutrient supplement containing diosgenin	Unclear	Various conditions	Therapeutic potential demonstrated	Unclear	Lipovac et al. (2016)
NCT06639698	Interventional Model : Single Group Assignment	Women with Polycystic Ovary Syndrome (PCOS) - Phenotype D	Diosgenin: 120 mg (Source: Dioscorea extract) Vitamin D: 50 mcg Alpha-Lactalbumin: 100 mg	6 Months	Restoration of Regular Menstrual Cycle	Active	Safety & Dosing of Diosgenin, but no relevance to OA	

## Conclusions and future directions

In this overall review, it is evident that diosgenin and other phytoconstituents have much to offer in the treatment of OA through various molecular pathways that occur to alleviate pain, inflammatory reactions, and cartilage degeneration. The multi-target nature of the compound is better compared to the conventional single target therapies since it is capable of inhibiting pro-inflammatory cytokines, NF- kB signaling, and maintaining extracellular matrix. Nevertheless, to achieve effective clinical translation, bioavailability obstacles should be overcome by innovative delivery strategies. These promising nano-formulations include targeted nanocarriers which can transform diosgenin into a treatment possibility that can be applied in clinical practice. The future research priorities should be Comprehensive clinical trials with optimized delivery formulations, Mechanistic research to elucidate tissue-specific effects and optimal dose regimens, Combination therapy models to exploit synergistic effects with existing therapies, Strategies to personalized medicine relying on genetic and inflammatory biomarkers and Reliable supply chains to enable clinical development using sustainable production methods.

Diosgenin-based therapeutics represent an attractive next-generation treatment option for the millions of individuals with OA due to the combination of natural product pharmacology, advanced drug delivery technology and precision medicine strategy.

Given the escalating global burden of OA and the toxicity limitations of current long-term therapies, there is a critical urgency to accelerate the transition of these preclinical findings into human trials. Future investigations must prioritise rigorous, randomised controlled trials designed to validate these novel delivery systems. Specifically, studies should propose evaluating the non-inferiority of diosgenin-loaded nano formulations compared to standard topical NSAIDs, with a focus on deep-tissue bioavailability and pain indices. Furthermore, longitudinal designs utilising intra-articular targeted nanocarriers are proposed to assess sustained release kinetics and cartilage preservation compared to conventional corticosteroid injections, thereby firmly establishing the clinical utility of this pleiotropic agent.

Future studies should focus on the following areas to bridge the gap between success in preclinical and clinical practice.

Advanced Clinical Trials: Studies should shift to the advanced randomised controlled trials (RCTs) that would authenticate these new methods of delivery. In particular, we suggest assessing the non-inferiority between diosgenin-loaded novel drug delivery systems and regular topical NSAIDs (e.g., diclofenac), with the deep-tissue bioavailability of diosgenin and standardised pain indexes (WOMAC/VAS) as the primary parameters.

Longitudinal Studies: Sustained-release kinetics and long-term cartilage preservation require longitudinal studies involving the use of intra-articular, targeted nanocarriers, as opposed to traditional corticosteroid injections. This will help determine the longevity of the treatment and its potential as a disease-modifying osteoarthritis drug (DMOAD).

Mechanistic and Synergistic Models: The field of mechanistic studies needs additional investigation to clarify tissue-specific actions and determine optimal dose schedules. Additionally, combinations of therapies need to be examined to utilise their synergistic actions with current therapies, and the required doses of synthetic anti-inflammatories may be reduced.

Precision Medicine: The development of personalised medicine strategies based on genetic and inflammatory biomarkers is necessary to detect subgroups of patients likely to respond to NF-6β pathway intervention.

Sustainable Production: To facilitate large-scale clinical development, it is essential to establish reliable supply chains and sustainable extraction methods.

## Data Availability

No datasets were generated or analysed during the current study.

## Declarations

### Generative AI and AI-assisted technologies in the manuscript preparation process

During the preparation of this work, the author(s) used Grammarly, and QuillBot to support language refinement, grammar correction, and improvement of sentence clarity and readability. After using these tools, the author(s) reviewed, edited, and validated all content manually to ensure accuracy, originality, and integrity of the final manuscript. The author(s) take full responsibility for the content of the published article.
